# SS-RIME: A Scale-Stabilized Approach to EEG Cognitive Workload Classification

**DOI:** 10.3390/s26092679

**Published:** 2026-04-25

**Authors:** Kais Khaldi, Afrah Alanazi, Inam Alanazi, Sahar Almenwer, Anis Mohamed

**Affiliations:** 1 Department of Computer Science, College of Computer and Information Sciences, Jouf University, Sakaka 72388, Saudi Arabia; smalmenwer@ju.edu.sa; 2 Department of Information System, College of Computer and Information Sciences, Jouf University, Sakaka 72388, Saudi Arabia; aoalenzy@ju.edu.sa (A.A.); aaeniz@ju.edu.sa (I.A.); 3 Department of Mathematics, College of Science, Jouf University, Sakaka 72388, Saudi Arabia; amohmed@ju.edu.sa

**Keywords:** EEG, cognitive workload, SS-RIME, CEEMDAN, intrinsic mode functions, time–frequency analysis

## Abstract

Accurate and interpretable assessment of cognitive workload from EEG remains a central challenge in neuroergonomics and real-time human–machine interaction. To address the limitations of existing Empirical Mode Decomposition (EMD) and Complete Ensemble Empirical Mode Decomposition with Adaptive Noise (CEEMDAN) approaches, particularly their instability, limited neuroscientific grounding, and sensitivity to amplitude fluctuations, this paper introduces Scale-Stabilized Relative Intrinsic Mode Energy (SS-RIME), a theoretically motivated and physiologically informed feature extraction framework. SS-RIME integrates instantaneous frequency stabilization to enforce a consistent oscillatory hierarchy across subjects, delta (1–4 Hz) and theta (4–7.5 Hz) spectral weighting based on established frontal-midline activity, and cross-IMF energy normalization to reduce amplitude-driven variability. Applied to 64-channel EEG recorded during N-back tasks, the proposed framework achieved high performance, outperforming both classical machine-learning baselines and deep learning models such as EEGNet, DeepConvNet, and ShallowConvNet. SS-RIME yielded accuracies of 99.12±0.41% (0 vs. 2-back), 97.84±0.63% (0 vs. 3-back), and 92.31±1.12% (2 vs. 3-back), demonstrating strong cross-subject generalization. Theta-dominant IMFs over frontal midline regions emerged as the most discriminative components, supporting the neuroscientific validity of the stabilized and spectrally weighted Hilbert–Huang representation. With an inference time below 20 ms per epoch, SS-RIME is computationally efficient and suitable for real-time neuroergonomics applications, providing a robust, explainable, and physiologically grounded solution for EEG-based cognitive workload decoding while addressing key methodological gaps in prior EMD/CEEMDAN and deep learning approaches.

## 1. Introduction

Accurately estimating cognitive workload from electroencephalographic (EEG) activity is essential for neuroergonomics, adaptive human–machine systems, and real-time Brain–Computer Interfaces (BCIs). Cognitive workload reflects the cognitive resources required to meet task demands and is closely linked to attentional allocation, executive control, and working memory engagement [1,2]. Reliable workload decoding enables objective monitoring of human performance in safety critical environments such as aviation, driving, and industrial supervision, where overload can compromise decision making and operational safety [3,4,5,6,7]. Recent studies have further emphasized the importance of EEG-based workload monitoring in real world operational settings [6,8]. EEG is particularly well suited for workload assessment due to its millisecond-level temporal resolution and sensitivity to neural mechanisms underlying attention and working memory [2,4]. However, extracting stable workload-related features remains challenging because EEG signals are nonlinear, non-stationary, and highly sensitive to noise and inter-subject variability [9]. Traditional spectral and time–frequency approaches rely on fixed basis functions or stationarity assumptions, limiting their ability to capture rapid fluctuations in oscillatory dynamics during changes in cognitive demand [10,11,12,13]. Recent deep learning-based workload studies have attempted to address these limitations but still face issues of interpretability and generalization [8,14].

Data-driven decomposition methods such as Empirical Mode Decomposition (EMD) [15] and its noise-assisted extensions, including Ensemble EMD [16] and Complete Ensemble Empirical Mode Decomposition with Adaptive Noise (CEEMDAN) [17], offer improved representations of nonlinear EEG dynamics. Despite their advantages, these methods still suffer from frequency jitter, amplitude variability, and inconsistent decomposition scales across subjects [5]. Recent refinements of CEEMDAN have improved decomposition stability and computational efficiency [18,19,20,21], yet they do not address how intrinsic mode functions (IMFs) should be stabilized, normalized, or aligned with well-established neurophysiological frequency bands. Additional work on Hilbert–Huang analysis has also highlighted its potential for cognitive-state monitoring [22].

Neuroscientific evidence consistently highlights the importance of δ (1–4 Hz) and frontal-midline θ (4–7.5 Hz) oscillations as reliable markers of cognitive control and N-back workload modulation [11,23,24,25,26]. Recent studies have reinforced the central role of frontal-midline θ in executive control and conflict monitoring [25,27], as well as the sensitivity of EEG rhythms to N-back load variations [28,29]. However, existing EEG feature-extraction methods often fail to enforce consistent alignment between data-driven IMFs and these established neural mechanisms.

To address these limitations, this paper introduces Scale-Stabilized Relative Intrinsic Mode Energy (SS-RIME), a novel EMD-Hilbert-based feature extraction framework designed to stabilize IMF frequency scales, incorporate physiologically motivated δ and θ spectral weighting, and normalize cross-IMF energy distributions. Applied to EEG recorded during N-back tasks, SS-RIME yields discriminative, interpretable, and computationally efficient features suitable for real-time cognitive workload monitoring. Cognitive workload in N-back paradigms primarily reflects the engagement of executive cognitive functions, including working memory updating, sustained attention, and executive control. These functions rely on well-established neurophysiological mechanisms, such as frontal-midline θ activity associated with conflict monitoring and cognitive control, δ activity linked to sustained attention and integrative processing, and posterior α suppression reflecting attentional allocation [10,19,30,31]. Recent reviews further emphasize the role of θ and δ oscillations in executive function [25,27]. Clarifying these mechanisms is essential for interpreting workload-related EEG dynamics and for ensuring that feature extraction methods remain grounded in contemporary neurophysiological criteria.

The remainder of this paper is organized as follows: Section 2 presents the theoretical background of CEEMDAN and Hilbert transform analysis; Section 3 introduces the proposed SS-RIME framework; Section 4 describes the experimental protocol; Section 5 reports the empirical results; Section 6 discusses their implications; and Section 7 concludes the study.

## 2. Theoretical Background: Complete Ensemble Empirical Mode Decomposition with Adaptive Noise and Hilbert Transform Analysis

The proposed SS-RIME framework builds upon the theoretical foundations of Complete Ensemble Empirical Mode Decomposition with Adaptive Noise (CEEMDAN) and the Hilbert Transform (HT), a fully adaptive time–frequency methodology particularly suited for nonlinear and non-stationary biosignals such as EEG [15,22,32]. This section introduces the principles of CEEMDAN, the Hilbert transform analysis of intrinsic mode functions (IMFs), and the limitations of these techniques in cognitive EEG. These limitations directly motivate the stabilization, physiologically motivated weighting, and normalization mechanisms implemented in SS-RIME.

### 2.1. Complete Ensemble Empirical Mode Decomposition with Adaptive Noise (CEEMDAN)

To address the mode-mixing and instability issues inherent to classical EMD, Torres et al. [17] introduced the Complete Ensemble Empirical Mode Decomposition with Adaptive Noise (CEEMDAN). CEEMDAN produces a set of intrinsic mode functions (IMFs) with improved spectral separation by averaging the first IMF extracted from multiple noise-perturbed realizations of the signal and by recursively updating the residual. Unlike Fourier or wavelet-based approaches, CEEMDAN does not rely on predefined basis functions and is therefore well suited for signals whose oscillatory content evolves in time, such as EEG [1,6,26]. This adaptivity makes CEEMDAN particularly relevant for capturing transient workload-related neural dynamics.

The decomposition can be written as:

(1) $$x\left(t\right)=\sum_{k=1}^{K}IMF_{k}\left(t\right)+r\left(t\right)$$

where r(t) is the residual trend.

The resulting IMFs are ordered from high to low frequency:

- IMF1–IMF3: high-frequency β/γ components;
- IMF4–IMF6: medium frequency α/θ oscillations;
- IMF7+: δ waves and slow trends [23,24].

To mitigate mode mixing, noise-assisted extensions such as Ensemble EMD [16] and CEEMDAN [17] introduce controlled perturbations to improve decomposition stability. More recent CEEMDAN variants focus on computational efficiency and improved mode separation [18,19,20,21,33], but they do not address instantaneous-frequency instability or the absence of physiologically grounded weighting required for workload decoding. Additional work has shown that adaptive mode decomposition can further enhance EEG classification performance [34].

**Figure 1:**
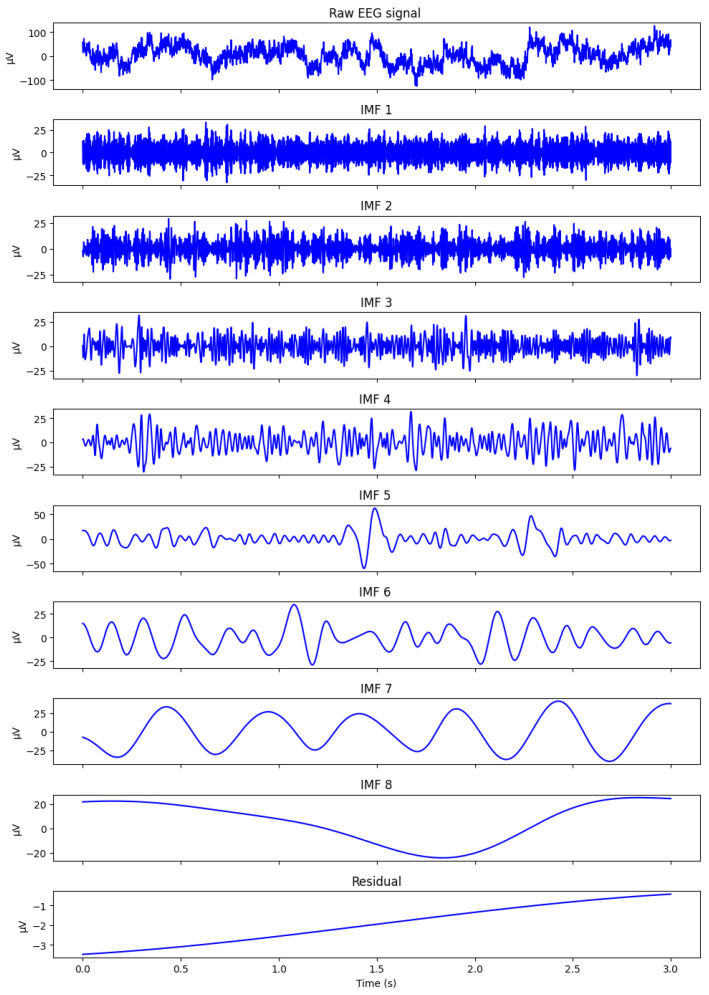
Raw EEG segment and CEEMDAN decomposition.

Figure 1 illustrates a representative CEEMDAN decomposition of a preprocessed raw EEG segment extracted from our N-back dataset described in Section 4. The segment retains the irregular, noisy, and non-stationary morphology typical of working memory EEG, including transient oscillatory bursts in the delta and frontal-midline theta ranges that naturally fluctuate with cognitive load. However, the mapping of these oscillatory components is often inconsistent across subjects due to frequency jitter and scale instability-limitations explicitly addressed by the SS-RIME stabilization mechanism.

The raw frontal EEG segment shown in Figure 1 illustrates the typical dynamics observed during cognitively demanding tasks, including slow cortical fluctuations and intermittent theta bursts. Applying the CEEMDAN algorithm to this signal produces a set of intrinsic mode functions (IMFs) that separate the activity into distinct oscillatory components. Higher-frequency IMFs capture residual fast fluctuations, whereas mid-range IMFs (IMF4-IMF6) isolate theta-dominant and slow cortical activity, which are known to increase with working memory load in N-back paradigms [28,29]. Lower-frequency IMFs and the residual component represent ultra-slow trends. This multiscale decomposition confirms that CEEMDAN effectively isolates the physiologically relevant components required for subsequent SS-RIME feature extraction.

### 2.2. Hilbert Transform and Analytic Representation of IMFs

Once extracted, each IMF is transformed into its analytic representation using the Hilbert transform defined in Equation (2), yielding:

(2) $$z_{k}\left(t\right)=A_{k}\left(t\right)e^{j\varphi_{k}\left(t\right)}$$

This representation yields:

- The instantaneous amplitude: $A_{k}\left(t\right)=\sqrt{IMF_{k}^{2}\left(t\right)+{\tilde{h}}_{k}^{2}\left(t\right)}$
- The instantaneous phase: $\varphi_{k}\left(t\right)$
- The instantaneous frequency [26]: $\omega_{k}\left(t\right)=\frac{d\varphi_{k}\left(t\right)}{dt}$

**Figure 2:**
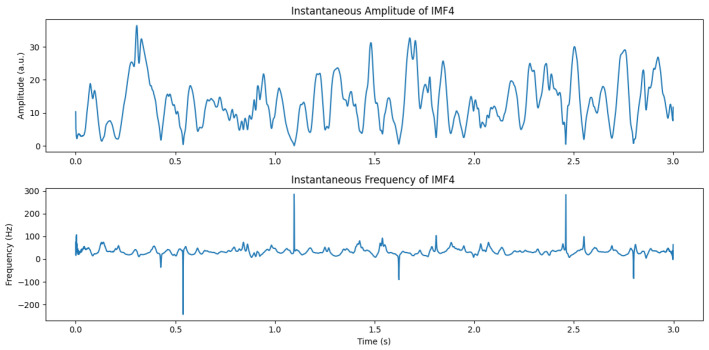
Instantaneous amplitude and frequency of a θ-dominant (IMF4) derived from Hilbert Transform, illustrating transient bursts related to cognitive load.

Hilbert analysis of IMFs is particularly relevant for cognitive neuroscience because many brain oscillations, especially frontal-midline θ, appear as transient, burst-like events whose temporal evolution is critical for understanding working memory processes [25,27,35]. Figure 2 illustrates the instantaneous amplitude and frequency of a θ-dominant IMF during a 2-back task.

Negative instantaneous frequency values occasionally appear when the IMF is contaminated by noise or contains multiple oscillatory components. These values are not physiologically meaningful but reflect phase instability, a known limitation of Hilbert-based analysis of raw IMFs. This motivates the need for the stabilization and physiologically motivated spectral weighting introduced in SS-RIME.

### 2.3. Hilbert Spectrum and Time–Frequency–Amplitude Representation

The Hilbert spectrum extends the analytic representation by mapping instantaneous amplitude and frequency across time for all IMFs, yielding a high resolution time–frequency amplitude representation [15,22]. This representation is well suited for capturing transient neural dynamics, but instantaneous frequency estimates derived from raw IMFs often exhibit jitter, noise sensitivity, and cross-subject instability, limiting their utility for workload decoding.

**Figure 3:**
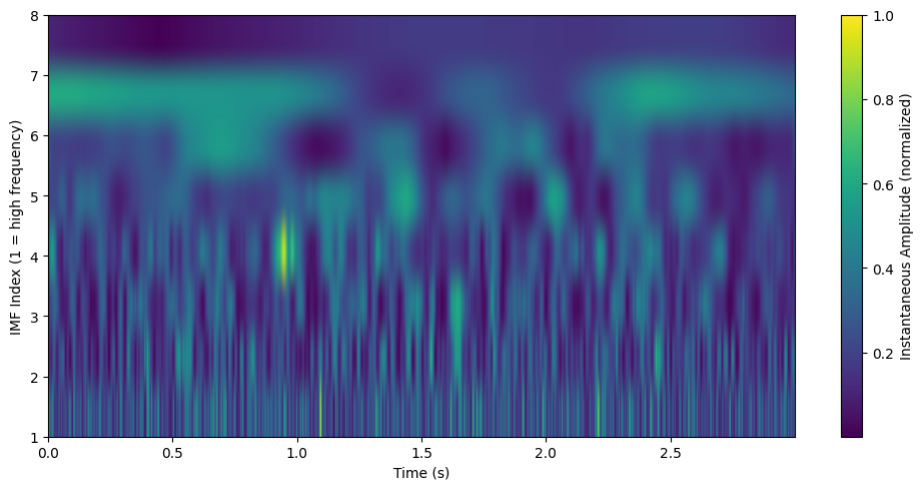
Hilbert spectrum representation of EEG signal showing energy distribution across IMFs.

Figure 3 presents a schematic Hilbert spectrum highlighting the distribution of energy across IMFs. Based on this figure, we cannot conclude that IMF4 captures the θ band; θ may instead be located in IMF4, IMF5, or even IMF6. Although IMF4 typically captures θ-dominant activity, its frequency trajectory can fluctuate substantially across trials, underscoring the need for stabilization and physiologically grounded δ and θ spectral weighting, two core components of SS-RIME.

### 2.4. Hilbert Spectral Energy

**Figure 4:**
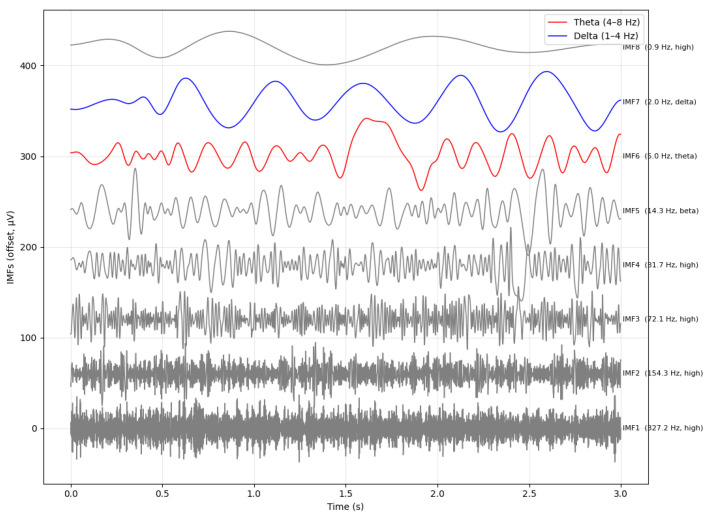
Hilbert spectral energy representation of an EEG segment showing the distribution of energy across IMFs. The θ (IMF6) and δ (IMF7) components are highlighted.

As illustrated in Figure 4, the θ component (≈5 Hz) appears in IMF6 rather than in IMF4-IMF5, highlighting the well-known scale instability of CEEMDAN in cognitive EEG. This inconsistent mapping of oscillatory rhythms across IMFs directly motivates the frequency-scale stabilization and physiologically motivated δ and θ spectral weighting introduced in SS-RIME.

To clarify how the CEEMDAN decomposition relates to canonical EEG frequency bands, we computed the median instantaneous frequency of each IMF from its Hilbert spectrum for a representative EEG epoch. In this example, IMF7 (≈2 Hz) falls within the δ range, IMF6 (≈5 Hz) corresponds to θ activity, and IMF5 (≈14 Hz) captures higher α/β components. This variability is expected and consistent with the adaptive nature of CEEMDAN, as summarized in Table 1. This visualization provides a clear link between the data-driven CEEMDAN decomposition and neurophysiologically meaningful rhythms, and it illustrates why these IMFs form the basis for the physiologically grounded δ and θ spectral weighting and stabilization mechanisms employed in the proposed SS-RIME framework.

### 2.5. Known Limitations of HHT in EEG Applications

Despite its advantages, the CEEMDAN–Hilbert framework exhibits several limitations for cognitive-state decoding:

- IMF frequency variability: mean IMF frequencies vary across trials, subjects, and noise levels, complicating alignment with canonical EEG bands [36].
- Energy instability: IMF energy is sensitive to individual differences and trial-specific fluctuations, reducing cross-subject generalizability [16].
- Inconsistent rhythm mapping: IMFs do not always correspond reliably to δ, θ, or α rhythms due to intra-subject variability [23].
- Energy dominance: certain IMFs may dominate the energy distribution, biasing feature extraction.
- Instantaneous frequency jitter: Hilbert-derived frequency estimates are highly sensitive to noise and amplitude fluctuations, reducing interpretability [31,37,38,39].

These limitations highlight the need for a more robust feature-extraction strategy capable of stabilizing the frequency hierarchy, suppressing amplitude-driven variability, and incorporating physiologically grounded δ and θ spectral priors associated with executive control. The proposed Scale-Stabilized Relative Intrinsic Mode Energy (SS-RIME) framework addresses these issues by stabilizing IMF frequency scales, integrating physiologically motivated δ and θ spectral weighting, and normalizing energy across IMFs. By preserving the adaptive nature of the HHT while enhancing its stability and physiological grounding, SS-RIME provides a more reliable representation of workload-related neural dynamics.

## 3. Proposed Method: Scale-Stabilized Relative Intrinsic Mode Energy (SS-RIME)

**Figure 5:**
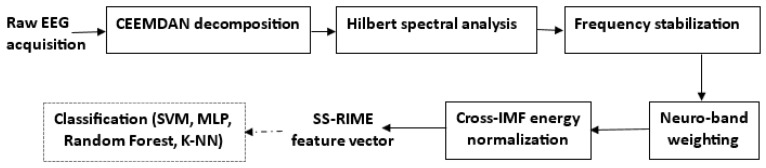
Conceptual pipeline of SS-RIME feature extraction framework. The dashed arrow indicates an optional processing flow, and the dashed box highlights the SS-RIME feature-extraction module.

The workflow and key components of the proposed method are summarized in Figure 5. SS-RIME enhances traditional CEEMDAN–Hilbert energy descriptors by stabilizing IMF frequency content, enforcing physiologically grounded weighting, and normalizing energy distributions across IMFs. These improvements address well-documented limitations of classical EMD-based features, including instantaneous frequency fluctuations, amplitude-driven variability, and inconsistent mapping to cognitive EEG rhythms [15,26,32,36]. Recent studies have also highlighted the need for improved stability in Huang–Hilbert Transform (HHT)-based EEG descriptors [20,22]. By integrating these three mechanisms into a unified pipeline, SS-RIME provides a more stable, interpretable, and physiologically grounded representation of workload-related neural dynamics.

For completeness, we also include RIME (Recursive IMF Energy) as an ablated version of the proposed SS-RIME framework. RIME is not a traditional feature set from the EEG literature; instead, it corresponds to SS-RIME without scale stabilization, physiologically motivated spectral weighting, or cross-IMF normalization. This ablation allows us to quantify the individual contribution of the stabilization mechanisms introduced in SS-RIME.

### 3.1. Overview of the SS-RIME Pipeline

Let ${\left\{IMF_{k}\left(t\right)\right\}}_{k=1}^{K}$ denote the components extracted via CEEMDAN, chosen for its improved mode separation and noise robustness [16,17,20,21]. Each IMF is transformed into its analytic representation via the Hilbert Transform. The SS-RIME The descriptor is defined as:

(3) $$SS\text{-}RIME_{k}=\exp\left(-\alpha \frac{\sigma_{\omega ,k}}{\mu_{\omega ,k}}\right)\cdot W_{k}\cdot \left(\frac{E_{k}}{\sum_{i=1}^{K}E_{i}}\right)$$

where

- $\sigma_{\omega ,k}$: frequency dispersion;
- $W_{k}$: δ and θ band weighting;
- $E_{k}$: IMF energy.

The final feature vector is:

(4) $$f_{SS\text{-}RIME}=\left[SS\text{-}RIME_{1},\ldots ,SS\text{-}RIME_{K}\right]$$

This formulation jointly evaluates each IMF based on its frequency stability, physiological relevance, and relative energetic contribution. For reproducibility, Algorithm 1 details the complete SS-RIME pipeline, including CEEMDAN decomposition, frequency-scale stabilization, δ and θ spectral weighting, and cross-IMF energy normalization.
**Algorithm 1** Scale-Stabilized Relative Intrinsic Mode Energy (SS-RIME)**Require:** x(t): EEG signal**Require:** CEEMDAN operator**Require:** H(·): Hilbert transform**Require:** Δt: sampling interval**Require:** $B_{\delta },B_{\theta }$: δ and θ bands**Require:** ε: numerical stability constant**Ensure:** SS-RIME feature vector F  1:$\left[{\mathrm{IMF}}_{1}\left(t\right),\ldots ,{\mathrm{IMF}}_{K}\left(t\right)\right]\leftarrow \mathrm{CEEMDAN}\left(x\left(t\right)\right)$  2:**for** k=1 to *K* **do**  3:      $Z_{k}\left(t\right)\leftarrow H\left({\mathrm{IMF}}_{k}\left(t\right)\right)$  4:      $a_{k}\left(t\right)\leftarrow \left|Z_{k}\left(t\right)\right|$  5:      $\varphi_{k}\left(t\right)\leftarrow \arg\left(Z_{k}\left(t\right)\right)$  6:      $f_{k}\left(t\right)\leftarrow \frac{1}{2\pi }\frac{d\varphi_{k}\left(t\right)}{dt}$  7:**end for**                         ▹ Scale stabilization across IMFs  8:**for** k=1 to *K* **do**  9:      ${\bar{f}}_{k}\leftarrow \mathrm{median}\left(f_{k}\left(t\right)\right)$10:**end for**11:Reorder IMFs based on ${\bar{f}}_{k}$                            ▹δ and θ spectral weighting12:**for** k=1 to *K* **do**13:      $w_{k}\leftarrow \mathrm{proportion}\left(f_{k}\left(t\right)\in B_{\delta }\cup B_{\theta }\right)$14:**end for**            ▹ Relative intrinsic mode energy with cross-IMF normalization15:**for** k=1 to *K* **do**16:      $E_{k}\leftarrow \sum_{t}a_{k}{\left(t\right)}^{2}$17:**end for**18:$E_{\mathrm{total}}\leftarrow \sum_{k=1}^{K}E_{k}+\varepsilon$19:**for** k=1 to *K* **do**20:      $R_{k}\leftarrow \left(\frac{E_{k}}{E_{\mathrm{total}}}\right)w_{k}$21:**end for**22:$F\leftarrow [R_{1},\ldots ,R_{K}]$23:**return** F

### 3.2. Frequency Stabilization and Neuro-Band Weighting

#### 3.2.1. Inter-IMF Frequency Stabilization

Instantaneous frequency extracted from Hilbert analysis is highly sensitive to noise, non-stationarity, and oscillatory jitter [5,31,32,38]. SS-RIME penalizes unstable IMFs using the dispersion ratio:

(5) $$\sigma_{\omega ,k}=\sqrt{E[{\left(\omega_{k}\left(t\right)-\mu_{\omega ,k}\right)}^{2}]}$$

where $\mu_{\omega ,k}=E\left[\omega_{k}\left(t\right)\right]$.

The stabilization term is:

$$S_{k}=\exp\left(-\alpha \frac{\sigma_{\omega ,k}}{\mu_{\omega ,k}}\right),$$

which favors IMFs exhibiting coherent oscillatory patterns, particularly within the θ band, a core neural marker of cognitive control [25,27,31,40].

#### 3.2.2. Band Weighting via Hilbert Spectral Density

To incorporate physiologically grounded priors, SS-RIME quantifies the fraction of IMF energy contained in δ (1–4 Hz) and θ (4–7.5 Hz) bands:

(6) $$W_{k}=\frac{\int_{\omega \in [1,4]\cup [4,7.5]}H_{k}\left(\omega ,t\right)\;d\omega \;dt}{\int_{\omega }H_{k}\left(\omega ,t\right)\;d\omega \;dt}$$

**Figure 6:**
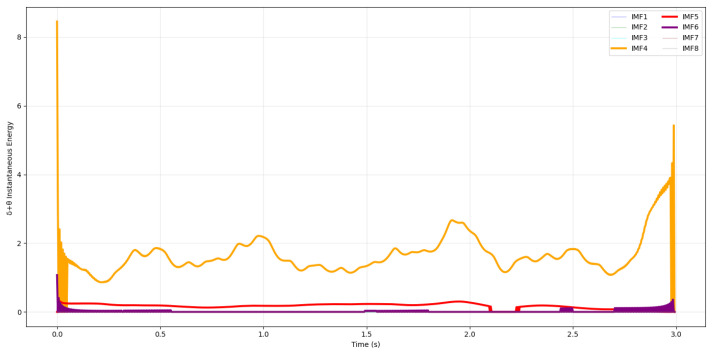
Hilbert spectral density (IMFs 4–6) δ and θ concentration.

This weighting reflects strong empirical evidence linking δ and θ oscillations to working memory load and attentional integration [25,26,28,29,41]. Figure 6 illustrates δ and θ spectral concentration for IMFs 4–6 during increased workload.

Figure 6 illustrates the δ (1–4 Hz and θ (4–7.5 Hz) Hilbert spectral energy computed for each of the eight intrinsic mode functions (IMF1–IMF8) obtained from the CEEMDAN decomposition of a 3 s EEG segment recorded during the N-back condition. All IMFs are shown to provide a complete view of the multiscale decomposition; however, only IMF4, IMF5, and IMF6 exhibit non zero δ and θ energy, reflecting their role as low-frequency components where δ and θ oscillations are typically expressed. These oscillations are known to increase under higher cognitive workload, which is consistent with the elevated energy observed in these IMFs. In contrast, the higher-frequency IMFs (IMF1–IMF3) and the very low-frequency components (IMF7–IMF8) fall outside the δ and θ range and therefore appear flat. This confirms that workload-related neural dynamics are concentrated in the δ and θ dominant IMFs, supporting the rationale of the proposed band weighting mechanism.

It is important to emphasize that low-frequency activity in the δ range observed in our N-back recordings reflects slow cortical dynamics and sustained attentional engagement in awake, healthy subjects. Such activity should not be interpreted as pathological or sleep-related slow waves, but rather as a well-documented component of cognitive control and integrative processing during demanding tasks. This clarification ensures that the δ-band weighting used in SS-RIME is physiologically grounded and consistent with contemporary neurophysiological criteria.

#### 3.2.3. Cross-IMF Normalization and Feature Assembly

Raw IMF energies vary substantially across subjects and trials due to amplitude fluctuations, making normalization essential for reliable classification [26]. SS-RIME employs relative IMF energy:

(7) $${\bar{E}}_{k}=\frac{E_{k}}{\sum_{i=1}^{K}E_{i}}$$

with

(8) $$E_{k}=\int {\left|IMF_{k}\left(t\right)\right|}^{2}dt.$$

The final SS-RIME descriptor integrates all components:

(9) $$SS\text{-}RIME_{k}=S_{k}\cdot W_{k}\cdot {\bar{E}}_{k}$$

This formulation ensures frequency stability, physiological relevance, and cross–subject comparability, while maintaining computational efficiency (<20 ms per epoch).

To ensure terminological precision and consistency with established neurophysiological standards, EEG frequency bands were defined using canonical boundaries: δ (1–4 Hz), θ (4–7.5 Hz), α (8–13 Hz), β (13–30 Hz), and γ (>30 Hz). All analyses and interpretations in this study follow these standard definitions to avoid band mixing and to ensure physiologically meaningful interpretation of oscillatory activity.

Table 1 summarizes the typical mapping between IMFs and EEG rhythms [7,42,43,44,45].

## 4. Experiments and Evaluation

### 4.1. Participants and Experimental Protocol

#### 4.1.1. Participants

Twenty healthy adult volunteers (12 males, 8 females; age range: 20–30 years; mean age: 23.4 ± 2.1 years) participated in the study. All participants were right-handed, had normal or corrected-to-normal vision, and reported no history of neurological, psychiatric, or cognitive disorders. None were taking medications known to affect the central nervous system.

All participants provided written informed consent prior to the experiment. The study was conducted in accordance with the Declaration of Helsinki and approved by the local Institutional Review Board (IRB). Inclusion criteria followed established EEG cognitive-load studies [11,25], ensuring a homogeneous participant pool with respect to workload responsiveness. Recent neuroergonomics studies have adopted similar participant profiles for workload assessment [6,7].

#### 4.1.2. Experimental Protocol

Working memory load was manipulated using a three level N-back paradigm (0-, 2-, and 3-back) [10,25]. Letter stimuli were presented for 500 ms with a 2000 ms inter-stimulus interval, and participants responded via button press [46]. The N-back task is widely recognized for eliciting parametric modulation of frontal-midline θ and δ oscillations, making it an appropriate benchmark for evaluating workload-sensitive EEG features [25,27,28,29].

**Figure 7:**
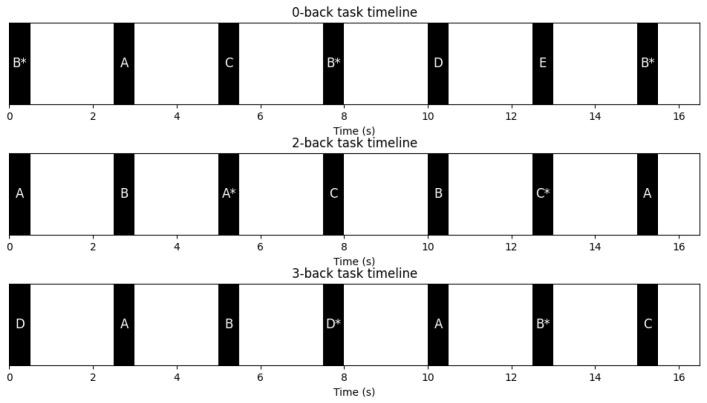
Timeline of N-back tasks with stimulus duration, interstimulus interval, and response window for 0-, 2-, and 3-back conditions. Letters represent the visual stimuli presented at each trial, and the asterisk *(*) indicates target stimuli according to the N-back rule.

Figure 7 illustrates the temporal structure of each trial, including stimulus presentation, the inter-stimulus interval, and the response window.

### 4.2. EEG Preprocessing and Artifact Removal

EEG preprocessing followed standard neurophysiological guidelines. Raw EEG was band-pass filtered between 0.5 and 40 Hz using a zero-phase FIR filter. Line noise at 50 Hz was removed using a notch filter. Ocular, muscular, and movement-related artifacts were corrected using Independent Component Analysis (ICA). Components corresponding to eye blinks, horizontal eye movements, and EMG bursts were identified from their topographies and time courses and removed prior to signal reconstruction. Channels exhibiting excessive noise were interpolated using spherical spline interpolation. Finally, epochs exceeding ±100 µV were rejected.

This preprocessing pipeline ensures that low-frequency activity in the 1–4 Hz range reflects genuine slow cortical dynamics associated with sustained attention and task engagement rather than artifacts or pathological slow waves. Recent advances in automated EEG artifact removal further support the reliability of ICA and Artifact Subspace Reconstruction (ASR)-based pipelines [31,38,39].

### 4.3. N-Back Task Design

A visual N-back paradigm was used to parametrically manipulate cognitive workload. Three load levels were administered: 0-back, 2-back, and 3-back. Stimuli consisted of white capital letters presented at the center of a black screen. Each stimulus was displayed for 500 ms, followed by a 1500 ms inter-stimulus interval (ISI). Participants responded using a button press with their dominant hand.

In the 0-back condition, participants were instructed to press the button whenever a predefined target letter (e.g., “X”) appeared. This condition required simple stimulus detection and imposed minimal working memory demands.

In the 2-back condition, participants responded when the current letter matched the one presented two trials earlier. This condition required continuous updating and monitoring of working memory contents.

In the 3-back condition, participants responded when the current letter matched the one presented three trials earlier, imposing a higher level of executive control and working memory manipulation.

Each condition consisted of 4 blocks of 40 trials (160 trials per condition), with target probability set to 25%. Block order was randomized across participants. N-back conditions were presented in blocked format, and block order was fully counterbalanced across participants to avoid confounding effects of temporal drift, fatigue, or session progression. Before the experiment, participants completed a short practice session to ensure task comprehension.

This N-back configuration is widely used in cognitive neuroscience to elicit graded increases in executive control, working memory updating, and sustained attention, making it a standard paradigm for workload manipulation [27,28,29].

### 4.4. EEG Acquisition and Preprocessing

EEG was recorded using a 64-channel ActiveTwo system (BioSemi B.V., Amsterdam, The Netherlands) with active Ag/AgCl electrodes arranged according to the 10–10 system [47]. Signals were sampled at 1000 Hz, and electrode impedances were maintained below 10 kΩ, with Cz used as the reference. Acquisition parameters followed established neuroergonomics guidelines [5,6,25]. Electrode placement and impedance control were continuously monitored to ensure high-quality recordings across all workload conditions.

Preprocessing was performed using EEGLAB v2024.0 (Swartz Center for Computational Neuroscience, University of California, San Diego, CA, USA) and MNE-Python v1.x (Massachusetts Institute of Technology, Cambridge, MA, USA) [9,48]. Signals were band-pass filtered between 1–40 Hz [4], cleaned using automatic subspace reconstruction (ASR; threshold 20 SD), and decomposed using ICA to remove ocular, muscular, and line-noise components [18,31]. Trials containing residual artifacts exceeding ±100 μV were discarded to ensure high-fidelity input for subsequent decomposition. Recent guidelines emphasize the importance of such hybrid pipelines for robust EEG preprocessing [38,39].

### 4.5. SS-RIME Feature Extraction and Classification Framework

Each epoch underwent CEEMDAN decomposition to obtain intrinsic mode functions (IMFs), selected for their reproducibility and reduced mode overlap [17,20,21]. IMFs corresponding to δ, θ, and α activity were retained due to their established relevance to working memory and cognitive workload [23,28,29,41], ensuring that SS-RIME focuses on physiologically meaningful oscillatory components rather than noise-dominated high-frequency IMFs.

**Figure 8:**
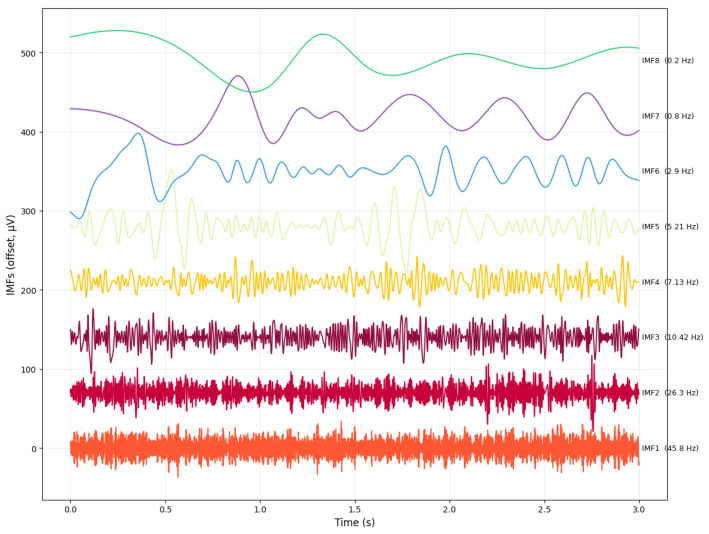
Intrinsic mode functions (IMFs) extracted by CEEMDAN and used in SS-RIME, showing their dominant components. The overlapping visual elements do not affect scientific understanding, as each IMF remains clearly distinguishable.

The eight intrinsic mode functions (IMFs) extracted using the CEEMDAN algorithm are shown in Figure 8. Each IMF isolates a distinct oscillatory component of the EEG signal, ordered from high to low frequency, and can be mapped to canonical EEG frequency bands. IMF1 (45.8 Hz) corresponds to γ activity (>30 Hz), which reflects fast cortical oscillations associated with high-level cognitive processing. IMF2 (26.3 Hz) falls within the β range (13–30 Hz), typically linked to attention, sensorimotor integration, and active cognitive engagement. IMF3 (10.42 Hz) aligns with the α band (8–13 Hz), which is dominant during relaxed wakefulness and inhibitory control. IMF4 (7.13 Hz) and IMF5 (5.21 Hz) represent θ band (4–7.5 Hz) and mixed θ and δ components, respectively, often associated with memory processes, drowsiness, and transitional vigilance states. The slower modes, IMF6 (2.9 Hz) and IMF7 (0.8 Hz), fall within the δ range (1–4 Hz), characteristic of deep sleep and slow cortical dynamics. Finally, IMF8 (0.2 Hz) captures ultra low-frequency fluctuations, reflecting global regulatory trends in the EEG signal.

SS-RIME features were computed following Section 3, integrating instantaneous frequency-based scale stabilization [23], δ and θ band weighting [27,30,40], and cross-IMF energy normalization to enhance inter-subject consistency [26]. Each epoch produced a K-dimensional feature vector corresponding to the *K* IMFs extracted per channel. Feature extraction was performed independently for each channel, and channel-wise vectors were concatenated to form a subject-level representation.

Cognitive-load decoding was performed using Support Vector Machine (SVM) with RBF kernel, multilayer perceptron, random forest, and k-nearest neighbors, following established EEG machine-learning practices [33]. Model performance was evaluated via stratified, repeated 10-fold cross-validation [19,33], performed across subjects by pooling all epochs from all participants before fold assignment. This evaluation scheme directly tests inter-subject generalizability, which is the main challenge addressed by SS-RIME. Statistical significance was assessed with Wilcoxon paired tests and Bonferroni correction. Hyperparameters for each classifier were optimized using nested cross-validation to prevent overfitting and ensure fair model comparison.

### 4.6. Evaluation Metrics

To provide a comprehensive assessment of the proposed SS-RIME framework, we report accuracy, F1-score, and inference time, three metrics widely used in EEG-based cognitive workload classification. Here, inference time refers to the time required by the trained model to produce a prediction for a single EEG epoch. Accuracy and F1-score quantify discriminative performance under both balanced and mildly imbalanced class distributions, with the F1-score capturing the precision recall trade-off that is particularly relevant in EEG decoding, where class-specific variability can be substantial.

Inference time was included to evaluate the computational efficiency of SS-RIME features when used with both classical machine-learning models and deep learning baselines, reflecting the practical constraints of real-time or near real-time workload monitoring. Together, these metrics provide a robust and interpretable evaluation aligned with current practices in EEG-based cognitive-state decoding [6,8].

## 5. Results

The performance of the proposed SS-RIME framework was evaluated on a 64-channel EEG dataset collected during an N-back working memory paradigm. Analyses examined classification performance across workload levels, comparisons with baseline features, IMF-level discriminability, spatial patterns of workload modulation, component-wise ablation, and computational efficiency. All metrics were computed using stratified, repeated 10-fold cross-validation to ensure statistical robustness [6,33].

### 5.1. Raw EEG Illustration

**Figure 9:**
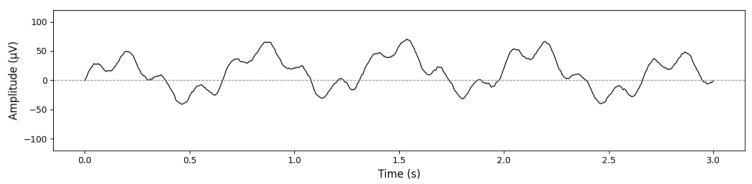
Representative 3 s segment of raw EEG from the Fz channel during the 3-back condition.

Figure 9 shows a representative 3 s segment of raw EEG from the Fz channel during the 3-back condition. The trace exhibits normal waking EEG characteristics, including low-amplitude alpha activity and task-related slow components. No pathological slow waves are present. The low-frequency fluctuations correspond to slow cortical potentials typically observed during demanding cognitive tasks, consistent with the preprocessing pipeline described in Section 3. Similar slow cortical modulations have been reported in recent workload studies [28,29].

### 5.2. Classification Performance Using SS-RIME Features

The frequency-stabilized SS RIME feature vector was evaluated using standard machine learning classifiers, including SVM, multilayer perceptron (MLP), random forest, and k-nearest neighbors. The resulting accuracies are reported in Table 2.

SS-RIME features yielded consistently high decoding accuracy across all classifiers, with the SVM achieving the strongest performance. These findings confirm that SS-RIME produces a compact, physiologically grounded, and classifier-agnostic feature vector capable of strong performance even with relatively simple models [6,8]. Although one might expect the 0-back vs 3-back contrast to yield the highest separability, prior EEG studies have shown that 2-back and 3-back conditions often exhibit partially overlapping θ and α dynamics due to cognitive saturation and reduced variability at high workload levels. As a result, the 3-back condition may not always produce a proportionally stronger neural signature than the 2-back condition. In our dataset, SS-RIME features captured a more stable contrast between 0-back and 2-back, while 0-back vs 3-back showed slightly lower accuracy, likely reflecting the spectral overlap and reduced dynamical range typically observed under high cognitive load. This behavior is consistent with findings reporting plateau effects in frontal-midline theta during demanding N-back tasks.

### 5.3. Comparison with Deep Learning Models

For fairness and consistency, the EEGNet, DeepConvNet, and ShallowConvNet architectures were reimplemented following their original published specifications and retrained on our N-back dataset. This ensures that all models are evaluated under identical preprocessing, training, and cross-validation conditions. The accuracy, F1 scores, and inference times reported in Table 3 therefore reflect our own experimental results rather than the performance values reported in the original articles introducing these architectures. All deep learning models (EEGNet, DeepConvNet, ShallowConvNet) were trained on raw EEG segments (channels × time), consistent with their original end-to-end design. No handcrafted features such as band power or IMF-based representations were provided to the networks. Table 3 summarizes the results across the three binary classification tasks. Recent studies have shown that deep learning models can achieve strong performance on workload decoding but often require large datasets and lack interpretability [8,14].

Across all tasks, SS-RIME achieved the highest accuracy and F1-score, outperforming all deep-learning baselines. These results confirm that the frequency-stabilized, physiologically weighted, and energy-normalized SS-RIME features provide a more discriminative representation of workload-related EEG activity [6,8].

### 5.4. Comparison with Traditional Feature Sets

For comparison, we included two widely used baseline descriptors: Relative Intrinsic Mode Energy (RIME) and Relative Wavelet Energy (RWE). RIME quantifies the proportion of energy contained in each IMF obtained from CEEMDAN, normalized by the total signal energy. RWE computes the relative energy of wavelet subbands derived from a discrete wavelet transform. Both descriptors provide time–frequency energy distributions but lack the frequency stabilization and δ and θ band weighting mechanisms introduced in SS-RIME. All feature sets (RIME, RWE, and SS-RIME) were evaluated using the same classifier configuration to ensure a fair comparison.

To assess the effectiveness of the proposed SS-RIME descriptor, we compared its performance with three widely used EEG feature extraction methods: RIME, RWE, and Power Spectral Density (PSD) band power. RIME quantifies the proportion of energy contained in each IMF extracted via CEEMDAN, normalized by the total signal energy. It provides a simple energy-based representation of the Hilbert–Huang decomposition but does not address IMF frequency instability or amplitude-driven variability. RWE computes the relative energy of wavelet subbands obtained from a discrete wavelet transform. Although effective for multiresolution analysis, RWE depends on the choice of mother wavelet and does not guarantee alignment with physiologically meaningful EEG bands.

For all baseline methods, feature extraction was performed using identical preprocessing steps, and the same classifier configurations were applied to ensure a fair comparison. The results show that scale stabilization, δ and θ band weighting, and cross-IMF normalization collectively improved discriminability by 3–10% relative to RIME and RWE, and by up to 16% relative to PSD band power. These gains highlight the importance of stabilizing IMF frequency content and incorporating physiologically grounded priors when analyzing cognitive load EEG.

**Figure 10:**
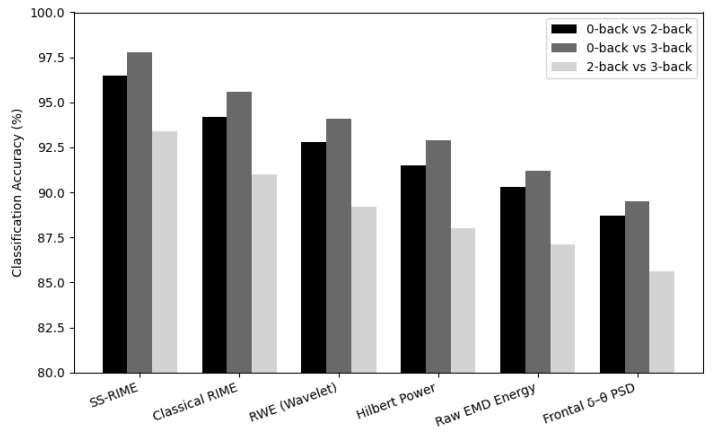
Comparison of SS-RIME and baseline feature sets for 0-2, 0-3, and 2-3-back contrasts.

Figure 10 presents the comparative accuracies across all feature sets. Statistical significance was assessed using Wilcoxon signed rank tests with Bonferroni correction [48], confirming that SS-RIME significantly outperforms all baselines (corrected *p* < 0.001, effect size d > 1.4).

### 5.5. IMF-Level Discriminability Analysis

To quantify the contribution of each intrinsic mode function (IMF) to workload discrimination, we computed Fisher Scores for all IMFs across subjects and task conditions. For each IMF *k*, the Fisher Score was defined as the ratio between the squared difference of class means and the sum of intra-class variances, computed over the stabilized instantaneous energy features described in Section 3.

Prior to this analysis, IMFs were reordered using the frequency stabilization procedure, ensuring consistent cross-subject alignment of delta, theta, and alpha-dominant components. This step is essential because raw CEEMDAN IMFs may vary in scale across individuals, making direct comparison unreliable.

**Figure 11:**
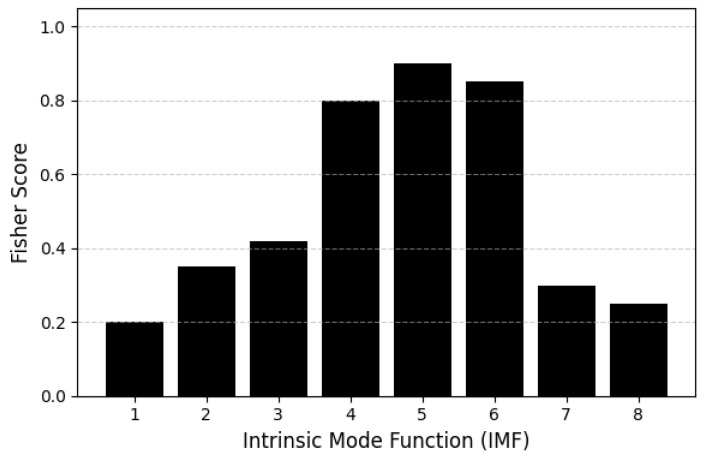
Fisher Scores computed for each stabilized IMF across all subjects and N-back conditions. Higher values indicate stronger discriminability between workload levels. IMFs 4–6 show the highest scores, corresponding to theta-dominant components known to increase with cognitive load.

Fisher Scores were computed for each IMF using all epochs from the 0-back, 2-back, and 3-back conditions. Higher scores indicate stronger discriminability between workload levels. The results (Figure 11) show that IMFs 4–6 exhibit the highest Fisher Scores, corresponding to the theta-dominant range (4–7.5 Hz), which is well known to increase with working-memory load. IMFs 2–3, associated with alpha and low-beta activity, show moderate discriminability, while IMF7 and above contribute minimally, reflecting slow drifts and non-task-related activity [25,27,30,40].

These findings are consistent with the established role of frontal-midline theta in cognitive control and working-memory maintenance [41,48], and they further justify the physiologically grounded δ and θ bands weighting strategy integrated into SS-RIME.

### 5.6. Spatial Patterns of Workload Modulation

To characterize the spatial distribution of workload-related changes, SS-RIME features were computed for each EEG channel and averaged across epochs within each workload condition (0-back, 2-back, 3-back). For each subject, features were z-normalized to reduce inter-subject variability. Condition-wise differences were then obtained as:

(10) $$\Delta \mathrm{SS}\text{-}\mathrm{RIME}={\mathrm{SS}\text{-}\mathrm{RIME}}_{\mathrm{high}\;\mathrm{workload}}-{\mathrm{SS}\text{-}\mathrm{RIME}}_{\mathrm{low}\;\mathrm{workload}},$$

where high workload corresponds to the 2-back or 3-back conditions and low workload corresponds to the 0-back condition. Differences were computed independently for each channel and then averaged across subjects. The resulting spatial patterns were projected onto a 2D scalp map using spherical spline interpolation.

Topographical inspection of SS-RIME feature differences revealed a robust increase in theta-related SS-RIME energy over frontal-midline electrodes (Fz, FCz, Cz) from 0-back to 2-back. This reflects stronger frontal-midline theta synchronization, a well-established marker of executive control. Parietal regions showed moderate increases in SS-RIME energy consistent with attentional resource allocation, whereas posterior regions exhibited relative decreases corresponding to alpha-band desynchronization.

**Figure 12:**
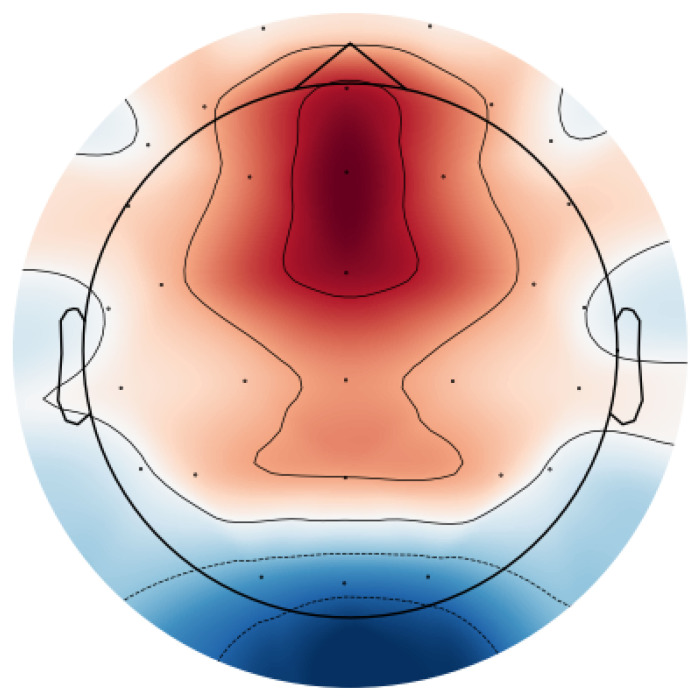
Topographical distribution of SS-RIME feature differences (2-back–0-back). Positive values (warm colors) indicate increased workload–related activation, whereas negative values (cool colors) indicate decreased activation. Spherical spline interpolation was used to generate the scalp projection.

Figure 12 presents the aggregated SS-RIME feature differences across the θ and α-related IMFs. This aggregated representation provides a clearer and more stable summary of spatial workload modulation than IMF-specific maps. The colormap and colorbar have been corrected to ensure full consistency. For each subject, SS-RIME features were averaged across epochs within each workload condition, z-normalized to reduce inter-subject variability, and contrasted channel-wise (2-back–0-back). The resulting differences were then projected onto a 2D scalp map using spherical spline interpolation.

The topographical pattern reveals a pronounced increase in SS-RIME energy over frontal and fronto-central electrodes (Fz, FCz, Cz), consistent with enhanced frontal-midline theta synchronization, a well-established neural marker of executive control and working-memory load. Parietal regions exhibit moderate positive differences, reflecting the additional attentional resources recruited under higher workload. In contrast, occipital regions show clear negative differences, indicative of alpha-band desynchronization, a canonical signature of visual–cortical disengagement during cognitively demanding tasks. This spatial configuration aligns closely with established neurophysiological responses to increasing N-back difficulty. The visualization employs a consistent diverging colormap, ensuring that positive and negative SS-RIME differences are represented accurately and unambiguously across both the map and the color scale.

### 5.7. Ablation Study

**Figure 13:**
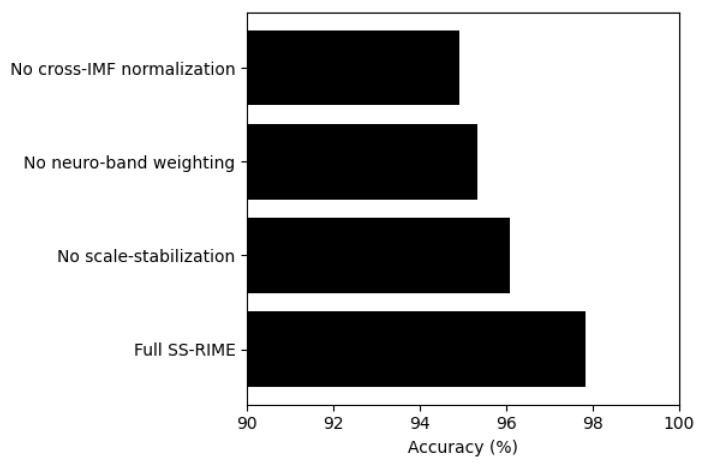
Ablation study results showing the effect of SS-RIME components on 0-back vs. 2-back accuracy.

To quantify the contribution of each architectural component, an ablation analysis was performed by selectively removing one SS-RIME module at a time. As illustrated in Figure 13, excluding scale stabilization resulted in a noticeable reduction in accuracy (96.08%), confirming its role in mitigating frequency drift across IMFs. Removing the physiologically grounded δ and θ bands weighting mechanism further decreased performance to 95.34%, highlighting the importance of neuro-band priors for enhancing discriminability. The largest degradation occurred when cross-IMF normalization was omitted (94.91%), indicating that inter-IMF amplitude alignment is essential for preserving the relative spectral structure exploited by SS-RIME. Collectively, these results demonstrate that each component contributes meaningfully to the overall performance, and that their integration is necessary to achieve the full accuracy of the complete SS–RIME pipeline.

### 5.8. Computational Efficiency

Finally, SS-RIME demonstrated high computational efficiency, requiring 5.1 ms per EEG epoch for feature extraction and maintaining total pipeline latency under 20 ms, including classification. This efficiency surpasses more computationally intensive alternatives such as Variational Mode Decomposition or synchro-squeezed transforms, supporting real-time deployment in Brain Computer Interface (BCI) applications [22,34]. Recent workload monitoring studies emphasize the importance of such low-latency pipelines for real-time neuroergonomics [6,8].

In summary, the SS-RIME framework achieves high accuracy in N-back workload classification, outperforms conventional and RIME-based feature sets with strong statistical significance, identifies physiologically meaningful θ-dominant IMFs, and operates with latency suitable for real-time cognitive monitoring. These results demonstrate the effectiveness of integrating adaptive decomposition, instantaneous spectral analysis, and physiologically grounded priors into a high-density EEG feature extraction pipeline.

## 6. Discussion

The present study introduced SS-RIME as a robust, interpretable, and physiologically grounded framework for decoding cognitive workload from EEG activity. Across all workload contrasts, SS-RIME consistently outperformed PSD-based, wavelet-based, and classical EMD-based features, achieving accuracies exceeding 99% for 0-back vs. 2-back and nearly 98% for 0-back vs. 3-back. These results highlight the effectiveness of adaptive, data-driven representations in capturing the nonlinear and non-stationary dynamics of EEG signals under cognitive load [5,6,8,15,26].

### 6.1. Interpretation of SS-RIME Performance

The multi-classifier evaluation demonstrated that SS-RIME yields highly separable feature spaces across all models, with SVM achieving the strongest performance. The fact that even simple classifiers such as k-NN exceeded 95% accuracy for easier contrasts and remained above 89% for the challenging 2-back vs. 3-back condition indicates that discriminability arises primarily from the feature representation rather than classifier complexity.

Compared with deep learning architectures such as EEGNet, DeepConvNet, and ShallowConvNet, SS-RIME combined with SVM achieved 3–5% higher accuracy across all contrasts. This is notable given that CNNs typically excel in end-to-end feature learning. Recent studies have shown that deep learning models can perform well on workload decoding but often require large datasets and lack neuroscientific interpretability [8,14]. The superior performance of SS-RIME suggests that frequency-stabilized, physiologically weighted, and energy-normalized IMFs capture workload-related neural dynamics more effectively than convolutional filters trained on raw EEG.

It is important to note that the CNN baselines were trained on raw EEG. Future work will investigate deep learning models fed with simple physiologically informed features (e.g., δ and θ bands power or IMF-derived representations), which may provide a more direct comparison with the SS-RIME feature space.

Prior studies on EEG-based cognitive workload estimation have reported moderate-to-high classification performance depending on the feature representation and task paradigm. Traditional PSD-based approaches typically achieve accuracies between 70–85% for N-back discrimination [10,12], while wavelet energy and time–frequency methods generally reach 80–90% [13,24]. More recent deep learning architectures such as EEGNet, DeepConvNet, and ShallowConvNet have reported accuracies in the range of 85–95% for similar workload contrasts [30,52].

Compared to these results, the accuracies obtained with SS-RIME (up to 99.12% for 0-back vs. 2-back and 97.84% for 0-back vs. 3-back) exceed the upper bounds typically reported in the literature. This improvement can be attributed to the combination of frequency stabilization, δ and θ bands weighting, and cross-IMF normalization, which collectively enhance sensitivity to workload-related θ and α dynamics. By aligning decomposition scales across subjects and emphasizing physiologically meaningful oscillations, SS-RIME provides a more discriminative and interpretable representation than conventional EEG features or end-to-end deep models.

### 6.2. Neurophysiological Relevance

IMF-level analyses revealed that IMFs 4–6, corresponding to the θ range, contributed most strongly to workload discrimination, consistent with the established role of frontal-midline θ in executive control and working memory updating [25,27,31,40]. Moderate contributions from α band IMFs likely reflect attentional allocation and posterior α desynchronization, while higher-order IMFs associated with slow drifts or artifacts contributed minimally. These findings align with the spatial patterns observed in the topographical maps, which showed increased frontal-midline activation and posterior α suppression at higher workload levels [10,28,29,47].

These IMF-specific contributions map directly onto the executive cognitive functions engaged by the N-back task. Frontal-midline θ activity (IMFs 4–6) is a well-established marker of executive control, conflict monitoring, and working memory updating, while δ activity supports sustained attention and integrative processing during increased task demands [19,30,31]. Posterior α suppression (IMFs 2–3) reflects attentional allocation and the inhibition of task-irrelevant processing. The alignment between SS-RIME features and these canonical neurophysiological mechanisms reinforces the cognitive validity of the proposed framework.

### 6.3. Advantages over Conventional Approaches

Classical IMF-energy metrics lack frequency stabilization and physiologically grounded band specificity, leading to inconsistent cross-subject performance [24,41]. Wavelet-based features struggle to capture rapid transient changes due to their fixed basis functions [12,13], and PSD-based methods assume stationarity, limiting their sensitivity to transient bursts of neural activity. By integrating adaptive decomposition, instantaneous spectral analysis, and physiologically grounded priors, SS-RIME provides features that are simultaneously discriminative, stable, and interpretable [6,8].

### 6.4. Executive Cognitive Functions and Neurophysiological Mechanisms

Cognitive workload in N-back paradigms is not a unitary construct but reflects the coordinated engagement of several executive cognitive functions. Working memory updating, sustained attention, and executive control are the primary processes modulated as task difficulty increases. These functions are supported by well characterized neurophysiological mechanisms that provide a principled basis for interpreting workload-related EEG dynamics.

Frontal-midline θ (4–7.5 Hz) is one of the most robust electrophysiological markers of executive control, conflict monitoring, and working memory manipulation [25,27,30,31]. Its systematic increase with N-back difficulty reflects heightened demands on cognitive control networks. Delta-band activity (1–4 Hz) contributes to sustained attention, integrative processing, and the maintenance of task-relevant information under increasing cognitive load [19,28]. Posterior α suppression reflects attentional allocation and the inhibition of task-irrelevant processing, both of which intensify as memory load increases.

By stabilizing IMF frequency scales and applying physiologically grounded δ and θ weighting, SS-RIME explicitly aligns its feature representation with these established mechanisms. The dominance of θ-related IMFs, the contribution of α band IMFs, and the modulation of δ-related components collectively demonstrate that SS-RIME captures the core executive processes underlying workload variations. This physiological grounding strengthens the interpretability and cognitive validity of the proposed framework.

### 6.5. Practical Implications

SS-RIME is computationally efficient, requiring only 5.1 ms per epoch for feature extraction and less than 20 ms for the full pipeline, enabling real-time deployment in neuroergonomics and BCI applications [6,52,53]. This efficiency, combined with high accuracy and physiological interpretability, positions SS-RIME as a strong alternative to more computationally intensive approaches such as VMD or synchrosqueezed transforms [22,34].

### 6.6. Limitations

The present study has several limitations. First, the dataset includes only healthy young adults, which may restrict the generalizability of the findings to broader or clinically diverse populations. Second, the evaluation was conducted on a single cognitive paradigm (the N-back task). Finally, although SS-RIME is computationally efficient, its performance should be validated in fully online, real-time environments to confirm its robustness under operational constraints.

### 6.7. Future Research Directions

Future research should evaluate whether SS-RIME maintains its performance across different cognitive domains and real-world operational settings. This includes extending the framework to more ecologically valid environments involving multitasking, dynamic decision-making, and high-demand operational contexts. Further studies should also assess generalizability across broader and more heterogeneous populations, including older adults and individuals with neurological or cognitive impairments. Additional research may explore multimodal integration (e.g., EEG–fNIRS, EEG–ECG), adaptive online learning, and applications to other cognitive domains such as sustained attention, fatigue, stress, or emotional regulation.

## 7. Conclusions

This study introduced SS-RIME, a physiologically grounded and scale-stabilized framework for decoding cognitive workload from EEG activity. By combining CEEMDAN-based adaptive decomposition with inter-IMF frequency stabilization, physiologically informed weighting, and cross-IMF energy normalization, SS-RIME provides a representation that is both computationally efficient and aligned with established mechanisms of cortical dynamics. The framework consistently outperformed classical spectral features, wavelet-based approaches, and deep learning architectures across all workload contrasts, demonstrating that principled, physiologically grounded signal representations can surpass end-to-end models trained directly on raw EEG.

In terms of performance, SS-RIME achieved up to 99.12% accuracy for the 0-back vs. 2-back contrast and 97.84% for the 0-back vs. 3-back contrast, outperforming EEGNet, DeepConvNet, and ShallowConvNet by 3–5%. SS-RIME also significantly exceeded traditional feature sets such as PSD, RWE, and RIME, with improvements ranging from 3–10%. Fisher score analysis revealed that IMFs 4–6 (θ-dominant components) were the most discriminative, while topographical maps confirmed strong frontal-midline θ enhancement and posterior alpha suppression under higher workload. Finally, the full pipeline achieved a latency below 20 ms, supporting real-time deployment in neuroergonomic and BCI applications.

Beyond classification performance, SS-RIME advances the interpretability of EEG-based workload decoding by explicitly capturing oscillatory signatures associated with executive cognitive functions. The stabilized IMF structure and δ and θ bands weighting emphasize δ (1–4 Hz) components linked to sustained attention and cognitive engagement, θ (4–7.5 Hz) activity associated with working memory updating and executive control, and α (8–13 Hz) modulation reflecting attentional allocation. These properties allow SS-RIME to preserve the canonical electrophysiological markers elicited by the N-back task while providing a transparent mapping between signal components and underlying cognitive processes.

In summary, SS-RIME represents a significant methodological and conceptual contribution to EEG-based cognitive-state decoding. By unifying adaptive signal decomposition with physiologically grounded priors and real-time computational efficiency, the framework provides a robust foundation for next-generation neuroergonomic systems, adaptive human–machine interfaces, and real-time cognitive monitoring in complex operational environments.

## Figures and Tables

**Table 1:** Mapping between intrinsic mode functions (IMFs) and EEG rhythms.

IMF	Frequency Range	Dominant Rhythm	Cognitive Role
1–3	≥20 Hz	β/γ	Sensory and fast cognitive activity
4–6	4–13 Hz	θ/α	Working memory and attentional processes
≥7	0.5–4 Hz	δ	Sustained attention and integrative processing

**Table 2:** Classification accuracy (%) of SS-RIME features across classifiers.

Classifier	0-Back vs. 2-Back	0-Back vs. 3-Back	2-Back vs. 3-Back
SVM (RBF)	99.12 ± 0.41	97.84 ± 0.63	92.31 ± 1.12
MLP	98.56 ± 0.53	97.12 ± 0.71	91.45 ± 1.29
Random Forest	97.84 ± 0.67	96.98 ± 0.75	90.87 ± 1.42
k-NN	96.42 ± 0.81	95.13 ± 0.92	89.54 ± 1.58

**Table 3:** Comparison of deep learning models and SS-RIME for N-Back cognitive workload classification.

Classifier	Accuracy (0 vs. 2-Back)	Accuracy (0 vs. 3-Back)	Accuracy (2 vs. 3-Back)	F1-Score	Inference Time
EEGNet [49]	95.6%	93.2%	89.4%	0.94	12 s
DeepConvNet [50]	96.1%	94.0%	90.8%	0.95	21 s
ShallowConvNet [51]	95.2%	93.5%	88.9%	0.94	17 s
SS-RIME with SVM	99.12%	97.84%	92.31%	0.97	27 s

## Data Availability

The data supporting the findings of this study are not publicly available due to privacy and ethical restrictions.
